# Charge transfer complex-based spectrophotometric analysis of famotidine in pure and pharmaceutical dosage forms

**DOI:** 10.1038/s41598-024-54402-4

**Published:** 2024-02-13

**Authors:** Shatha Y. Al Samarrai, Radhi AlZubaidi, Nadhir Al-Ansari

**Affiliations:** 1 Chemistry Department, College of Science, Tikrit University, Tikrit, Iraq; 2 Civil and Environmental Engineering, College of Engineering, University of Sharjah, Sharjah, United Arab Emirates; 3 Department of Civil, Environmental and Natural Resources Engineering, Lulea University of Technology, Luleå, Sweden

**Keywords:** Charge transfer complex, Famotidine, *Ortho-*chloranil, Spectrophotometric, Biochemistry, Environmental sciences

## Abstract

A straightforward and efficient spectrum technique was created using *Ortho-*chloranil as the electron acceptor (-acceptor) in a charge transfer (CT) complex formation reaction to determine the concentration of famotidine (FMD) in solutions. Compared to the double-distilled blank solution, the reaction result detected a definite violet colour at a maximum absorption wavelength of 546 nm, For concentrations range 2—28 µg/ml, the technique demonstrated excellent compliance with Beer-Law and Lambert's, as evidenced by its molar absorptivity of 2159.648 L mol^−1^ cm^–1^. Lower detection limits of 0.3024 µg/ml and 1.471 µg/ml, respectively, were discovered. The complexes of famotidine and *Ortho-*chloranil were found to have a 2:1 stoichiometry. Additionally, the suggested approach effectively estimated famotidine concentrations in pharmaceutical formulations, particularly in tablet form.

## Introduction

**Figure 1:**
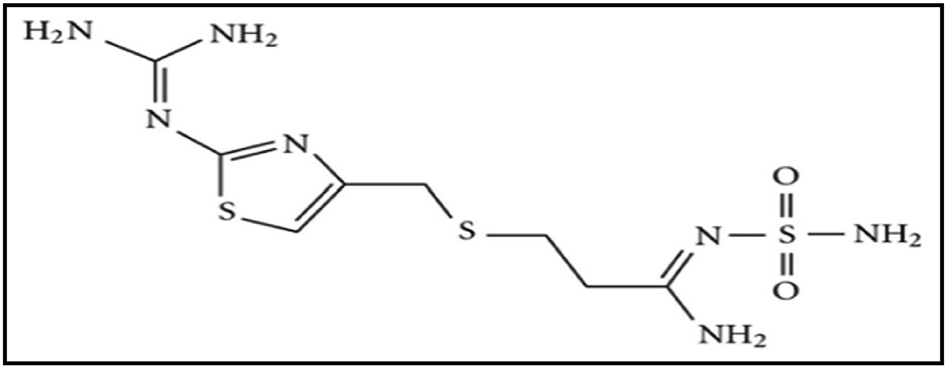
Famotidine's chemical composition.

Famotidine, scientifically referred to as 3-[2-(diaminomethyleneamino) thiazol-4-ylmethylthio]-N-sulfamoylpropionamidine, is a type of medication that blocks H_2_ receptors and acts as an antihistamine. It is commonly prescribed to treat stomach and duodenal ulcers, as well as gastric reflux disease^1–3^. The therapeutic dosage of famotidine is typically 40 mg administered daily, and it is absorbed completely Through the gastrointestinal tract, the highest concentration of the substance in the blood is reached around 3 h after it is taken orally^4,5^. Figure 1 shows the chemical structure of famotidine.

The literature has documented several methods for analyzing famotidine in pharmaceutical formulations and biological fluids. Spectrophotometric methods, as well as chromatographic methods, have been widely employed^6–8^. Additionally, the flow injection analysis method using spectrophotometry has been utilised^9,10^. Electrochemical methods such as the voltammetric method^11–13^ and the polarography method have been explored, along with the use of ion-selective electrodes^14,15^.

Due to issues with reaction time, sensitivity, selectivity, efficacy, economic cost, and high detection rates, many of these approaches, however, have limits for famotidine screening^16,17^. In contrast, the flow-injection analysis (FlA) technology has proven to be a successful and promising approach for famotidine analysis. It has benefits, including little handling of reagent samples, quick reaction times, good prediction rates, and reproducibility^18,19^. Additionally, FlA can evaluate colourful and turbid solutions^20^.

This work aims to determine a straightforward, sensitive, and precise spectrophotometric method to estimate famotidine in pharmaceutical formulations. The study will concentrate on ideal circumstances and elements influencing electrostability.

## Expermintal

### Devices

In this study, spectrophotometric measurements were carried out using an Japanese SHIMADZU UV–Visible-1800 double-beam spectrophotometer. Furthermore, pH (3310, Jenway) values ​​were measured, and sonicator (Labtech LUC-405, South Korea) and centrifuge (Hettich, Germany). A careful selection of this sophisticated equipment is intended to ensure the accuracy and reliability of data collection throughout the testing procedures.

### Reagents and chemicals

The present study used a famotidine (20 mg/tablet) formulation which was obtained from Samra Drug of Iraq (SDI), a pharmaceutical company that complies with British Pharmacopoeia standards in its formulation. Methanol (CAS number: 67–56-1; BDH; UK) is used as a solvent chemical in experimental procedures, while *Ortho-*chloranil (CAS number: 2435–53-2; Fisher Scientific, US) was a significant reagent. Additional chemical substances, including NaOH (CAS number: 1310–73-2; Kishida Chemical Co., Ltd. Japan), Cetyltrimethylammonium Bromide (CTAB), CAS number 57–09-0, and Sodium dodecyl sulfate (SDS), CAS number 151–21-3—that you purchased from Sigma-Aldrich® in Germany, The CAS number 9036–19-5 Triton X-100 was acquired from BDH in the United Kingdom, Na_2_CO_3_ (CAS number: 497–19-8) and KOH (CAS number: 1310–58-3) obtained from Merack, USA, and double-distilled water were used in this study.

#### Famotidine standard solution (100 µg.ml)

0.01g was dissolved using a small quantity of double-distilled water that created a solution. Subsequently, the volume in the 100 ml volumetric flask was incrementally raised to the intended level using the identical solvent.

#### *Solution for ortho-*chloranil *(0.005M)*

The Ortho-chloranil powder (0.123 g) was mixed with the amount of methanol. Following this, the volume in a 100 ml volumetric flask was carefully set to the mark.

#### Pharmaceutical solutions (tablets)

Ten tablets of 20 mg each were used in this study. FMD tablets were crushed into a fine powder, after which the equivalent of 0.1 g was prepared. The obtained powders were dissolved in a proportionate amount of double-distilled water. The dissolution process was performed by ultrasonication for 15 min and centrifuging for 5 min. The filtrate was separated using Whatman filter paper No. 41. The resulting mass was transferred to a100 ml volumetric flask, and the volume was completed to the mark.

#### Approximate NaOH solution (0.1M)

Dissolve 0.400 g of NaOH with the appropriate amount of double-distilled water in a 100 ml flask and completing to the mark.

#### Solution of surface active compounds (0.1%)

Dissolve 0.100 g of each surfactant compound CTAB and SDS and Triton X-100 were added to a 100 ml volumetric flask filled with hot double-distilled water to dissolve the compounds.

#### The general principle of the procedure

The method is mostly based on the interesting way that famotidine and *Ortho-*chloranil work together as π-acceptors. The absorbance at 546 nm shows that this interaction occurs in an alkaline medium compared to a double-distilled blank solution. For future studies and applications in this chemical system, this analysis tool acts as a source of vital information regarding the formation and quantification of these complexes.

## Results and discussion

### Optimisation conditions

1.3 ml of *Ortho*-chloranil at a concentration of 0.005 M, 0.5 ml of FMD at 100 μg/ml, and 0.5 ml of NaOH at a concentration of 0.1 M, respectively, in a 5 mL volumetric flask. The spectrophotometric readings were carefully made using the maximum absorption wavelength at 546 nm and compared to double-distilled blank solutions. The accuracy of experimental results is ensured by this careful procedure, so that it is reliable even further, making it possible to have a better understanding of how these compounds’ components interact with one another in detail.

### The influence of the base solution

A solution with a concentration of 0.1 M was used to make the alkaline solution. A fixed amount of 0.3 ml of each chemical was mixed with the famotidine solution, The absorbance was measured at 546 nm , and Table 1 shows the results.

**Table 1:** Base type effect.

Base type	Abs
NaOH	0.07
Na_2_CO_3_	0.02
KOH	0.03

Abs.: Absorbance.

As seen in Table 1, different bases affect famotidine dye absorption at 546 nm. The data indicates that NaOH displayed the most intense color, with an absorbance value of 0.07. When comparing, Na_2_CO_3_ and KOH, 0.02 and 0.03 were the respective low absorption values that were observed. Table 1 indicates that famotidine is strongly absorbed and reacts well with NaOH, making it the best choice for further research.

### Effect of sodium hydroxide volume

Varying amounts of NaOH have been studied to determine their ability for absorption by the FMD. The volumes ranged from 0.1 ml to 1.9 ml. They were added to a volumetric flask that already contained 0.5 ml of FMD solution with a concentration of 100 µg /ml, as well as 1 ml of *Ortho*-chloranil reagent solution with a concentration of 5.0 × 10^–3^ M. The final volume was decreased to 5 ml using double distilled water, as shown in Table 2.

**Table 2:** NaOH volume's effect.

NaOH volumes (ml)	Abs. (nm)
0.1	0.025
0.3	0.071
0.5	0.084
0.7	0.073
0.9	0.066
1.1	0.063
1.3	0.062
1.5	0.061
1.7	0.054
1.9	0.047

After doing data analysis, it was determined that the best concentration for FMD absorption, which attained its maximum value of 0.084, was 0.5 ml of sodium hydroxide (1 × 10^−1^M) at pH 10.5. Hence, this particular quantity of sodium hydroxide was considered ideal and then was utilized for further studies. The increased absorbance at this volume indicates a positive interaction between famotidine and the prescribed quantity of sodium hydroxide, which is an essential factor for obtaining dependable and precise outcomes in subsequent analyses and applications.

### Influence of the quantity of ortho-chloranil reagent

An investigation was conducted to examine the impact of different quantities of *Ortho-*chloranil reagent on the absorbance of the famotidine solution. Adding different amounts (0.1 ml to 1.9 ml) of a reagent solution with a concentration of 5 × 10^−3^M to volumetric flasks that already had 0.5 ml of a 100μg/ml famotidine solution was done. Afterwards, 0.5 ml of a solution containing 1 × 10^−1^M sodium hydroxide was introduced, and the total volume was modified to 5.0 ml by adding double-distilled water. These studies show how the *Ortho-*chloranil reagent's volume affects absorbance values in Table 3.

**Table 3:** Effect of *ortho-*chloranil reagent amount.

Volumes of *ortho-*chloranil (ml)	Abs
0.1	0.009
0.3	0.017
0.5	0.024
0.7	0.053
0.9	0.081
1	0.093
1.1	0.108
1.3	0.124
1.5	0.111
1.7	0.102
1.9	0.085

Data analysis revealed that famotidine absorbance peaked at 0.124 with 1.3 ml of *Ortho*-chloranil reagent (5 × 10^−3^M). The increased absorbance at this volume signifies a positive interaction between famotidine and the given quantity of *Ortho-*chloranil, giving it a critical parameter for obtaining dependable and precise outcomes in subsequent experiments and applications.

### Effect of surface-active substances

Different metabolites were tested to see how they affected famotidine-*Ortho*-chloral complex absorption.The study focused on three metabolites: SDS, Triton X-100, and CTAB. Investigations were carried out, According to Table 4, these surfactants reduced water absorption, making them unsuitable for future use.

**Table 4:** Substances' surface effect.

Surfactant 0.1% (1ml)	Abs. at 546 nm
Surfactant-free	0.124
SDS	0.117
Triton X-100	Turbid
CTAB	Turbid

Table 4 shows that the surfactant-free solution possessed the maximum absorption value, measuring at 0.124. Nevertheless, the use of SDS resulted in an average decrease in absorbance of 0.117. However, the use of Triton X-100 and CTAB led to a turbid solution, suggesting an important interference with the absorbance measurements.

They were determined to be unsuitable for further testing because of reduced absorption or contaminants resulting from their reactivity. The absence of synthesized surfactants yielded consistent outcomes that were particularly resilient for the famotidine- *Ortho-*chloranil complex, providing precise data acquisition and analysis in subsequent experimental investigations.

### Effect of temperature

An investigation was conducted to examine the impact of temperature (ranging from 15°C to 65°C) on the reaction of charge transfer complexes, using settings that were optimized. According to the findings, the rate at which absorption occurred changed depending on the temperature. Table 5 shows that absorption was maximum at 30 °C.

**Table 5:** Temperature effect.

Temp. (°C)	Abs
15	0.077
20	0.098
25	0.112
30	0.121
35	0.115
40	0.109
45	0.107
55	0.097
60	0.089
65	0.081

The data analysis revealed that the charge transfer complex had the highest level of adsorption at a temperature of 30° C. As a result, this temperature was regarded as appropriate and chosen for the following experiments. The absorbance values demonstrated a clear and consistent relationship with temperature. At elevated temperatures, the tensile strength indicated an initial increase until it exceeded 30 °C, after which it began to decrease. The result is highly valuable for comprehending the kinetics and thermodynamics associated with the formation of charge transfer complexes.

### Effect of order of addition

A series of extensive experiments were conducted to assess the impact of various modification strategies on the absorption properties of the violet material. The results obtained from the three distinct methodologies are thoroughly described and displayed in Table 6.

**Table 6:** Effect of order of addition.

Order of addition	Abs
Reag. + NaOH + Drug	0.09
Drug + Reag. + NaOH	0.122
Drug + NaOH + Reag	0.05

Upon comprehensive data analysis, it is evident that the additive structure has an important influence on the absorption of the violet material. The absorption level varies depending on the reagent (Reag.), NaOH, and drug that are added.

1. The absorbance was 0.09 after adding the reagent, NaOH, and drug subsequently.
2. When the drug, reagent, and NaOH were added in order, the absorbance increased to 0.122.
3. Lastly, the absorbance was comparatively lower at 0.05 when the medication was added first, then NaOH, and finally the reagent.

The results of the study demonstrate the significant effect of additive structure on the creation of violet products. As a result, the second combined system (Drug + Reag. + NaOH) had the highest absorption, indicating its preference for the following tests. An appropriate configuration of this setting is required.

### Effect of reaction time

The experiment attempted to examine the effects of time on the stability of the pigment complex that is created when the reactants are mixed under optimum experimental conditions. The stability of pigment absorption takes a minimum of 40 min, as seen in Table 7.

**Table 7:** Effect of time.

Time (min)	Abs
1	0.045
5	0.124
10	0.124
15	0.123
20	0.121
25	0.121
30	0.121
35	0.120
40	0.120
45	0.102
50	0.097
55	0.096
60	0.093

Data show that solid absorbance was stable and constant for at least 40 min after mixing the active ingredients, and the absorbance values were observed to be between 0.120 and 0.124 at this point, indicating that the composition remains available colors. However, after 40 min, the adsorption capacity decreased gradually, indicating a gradual decrease in color intensity.

The obtained result suggests that the color complexity, resulting from the combination of reaction components under optimum experimental conditions, remains constant for roughly 40 min. Experimental design and study reliability and consistency require this information when using complex color schemes in complex environments.

### Final absorption spectrum

**Figure 2:**
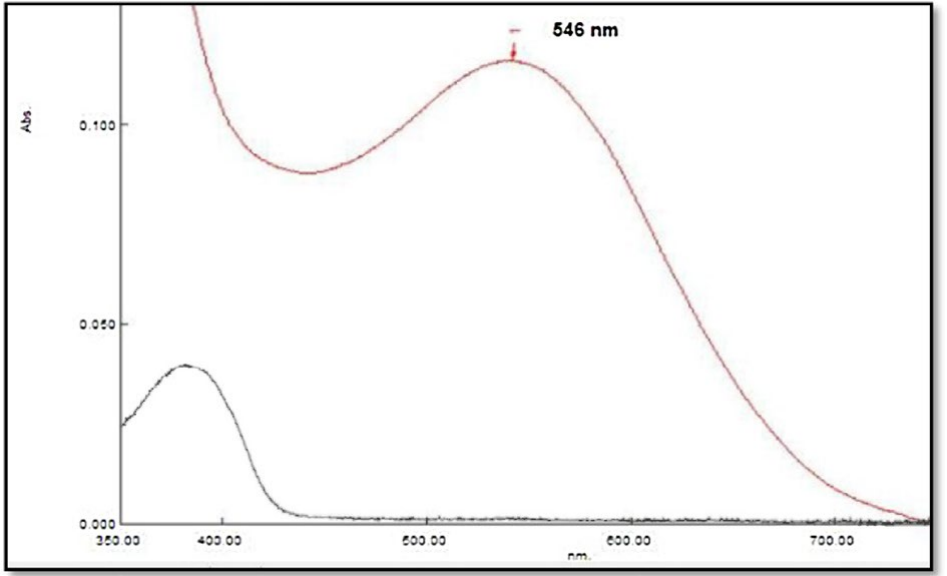
The absorption spectrum of FMD—*ortho-*chloranil complexes.

Synthesized colour material absorption spectrum was measured by combining 10 µg/ml of FMD with *Ortho-*chloranil reagent (5 × 10^−3^M) in a starting solution of 1 × 10^–1^ M NaOH at a pH of 10.5 and a temperature of 30 °C. Max absorbance (λ max) was measured at 546.0 nm. See Fig. 2.

According to the data, Table 8 shows the best FMD Ortho-chloranil complex creation conditions.

**Table 8:** Summary of optimum condition.

Experimental condition	
max (nm) λ	546
Amount (ml) of 0.1 M sodium hydroxide	0.5 ml
Amount (ml) of 5 × 10^−3^ M *Ortho-*chloranil reagent	1.3 ml
Temperature	30 °C
Solvent	Water
pH	10.5

The optimum conditions involve measuring the absorbance at 546 nm, using 0.5 ml of 0.1 M sodium hydroxide and 1.3 ml of 5 × 10^–3^ M *Ortho-*chloranil reagent. The temperature of the reaction should be maintained at 30 °C, and water serves as the solvent under a basic medium with a pH of 10.5. These optimal conditions ensure the highest sensitivity and stability of the formed colored complex, providing accurate and consistent results in further analytical studies involving the FMD—*Ortho-*chloranil complexes.

### Methodology for constructing the calibration curve

**Figure 3:**
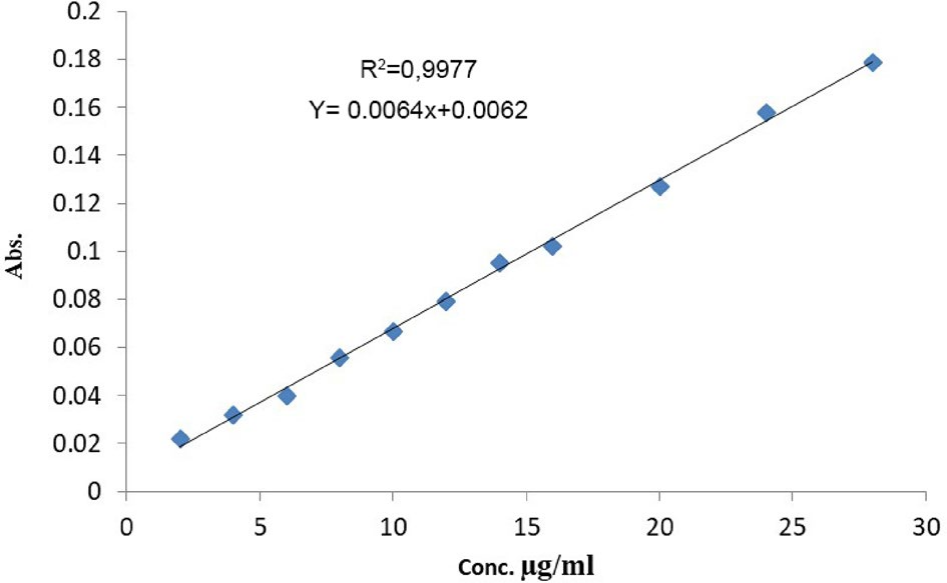
Famotidine calibration curve.

Add increasing amounts of FMD (100 μg/ml) to a series of solutions ranging from 0.1 ml to 2.0 ml. Combine these solutions with 1.3 ml of 0.005M *Ortho-*chloranil and 0.5 ml of 0.1 M NaOH in a 5.0 ml volumetric flask. Finally, fill the flask to the mark with distilled water and measure the absorption of all solutions versus a double-distilled blank solution at 546.0 nm. A linear calibration curve for FMD with a concentration of 2–28 μg/ml, R^2^ = 0.9977, a molar absorption coefficient of range 2159.648 /mol.cm, and a Sandell Index of 0.15625 μg/cm^2^, are shown in Fig. 3.

### Accuracy and precision

For each concentration of 100 μg/ml, the recovery percentage (Rec%) and standard deviation (RSD) were calculated to confirm these results. By taking an average of six readings for each, the recovery rate was 99.93%, and the relative standard deviation (0.781–0.928%) and results in Table 9 are shown, meaning that the method is highly accurate and has a good agreement.

**Table 9:** Precision and accuracy.

Conc. taken µg/ ml of famotidine	Conc. found µg/ ml of famotidine	Rec%	Average of Rec%	RSD%
4	4.129	103.225	100.345	0.928
12	11.871	98.71		0.781
20	19.951	99.10		0.972

Note: Rec%: Recovery; RSD%: Relative Standard deviation.

### Limit of detection (LOD) and limit of quantification (LOQ)

The limit of detection (LOD) was determined by assessing the absorption of the lowest concentration (2 μg/ml) in the calibration curve under the optimal conditions. The LOD and LOQ values of the suggested approach are presented in Table 10.

**Table 10:** Validation of optical characteristics and data using a spectrophotometric technique.

No	Parameters data	Data
1	λ max nm	546.0
2	Beers Law limit μg/ml	2.0—28
3	Colour	violet
4	Correlation Coefficient	0.9977
5	Sandell sensitivity μg/cm^2^	0.15625
6	Molar absorptivity L/ mol.cm	2159.648
7	Average of Rec%	100.345
8	LOD (μg/ml )	0.324 μg/ml
9	LOQ (μg/ml )	1.471 μg/ml

### The reaction's stoichiometry

**Figure 4:**
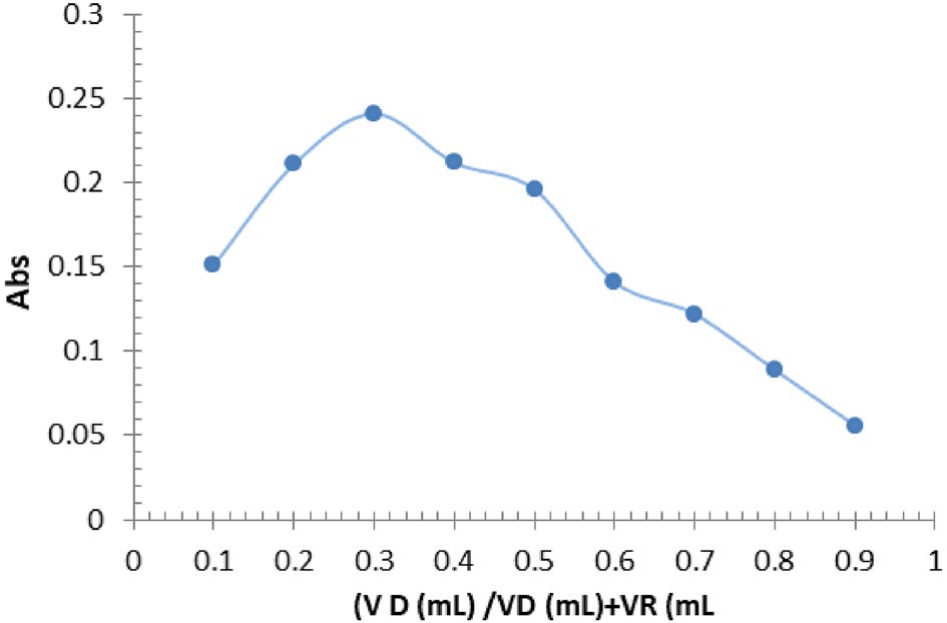
Job Methods.

The stoichiometry of the reaction between FMD and Ortho-chloranil is the quantitative relationship between the amounts of reactants and products involved in the chemical reaction. The concentration of the FMD and Ortho-chloranil was set at 5 × 10^–3^ M in both the Job's method and the molar ratio method, Used a set of volumetric flasks with a capacity of 5.0 ml to contain varying amounts of the drug solution ranging from 0.2 to 1.8 ml, as well as variable volumes of the reagent solution ranging from 1.8 to 0.2 ml. Added 0.5 ml of a 0.1 M NaOH solution, and then filled the flasks to the mark with double-distilled water. The absorbance was quantified at a wavelength of 546.0 nm relative to the blank reagent. The figures in Fig. 4 indicate a ratio of 2:1.

**Figure 5:**
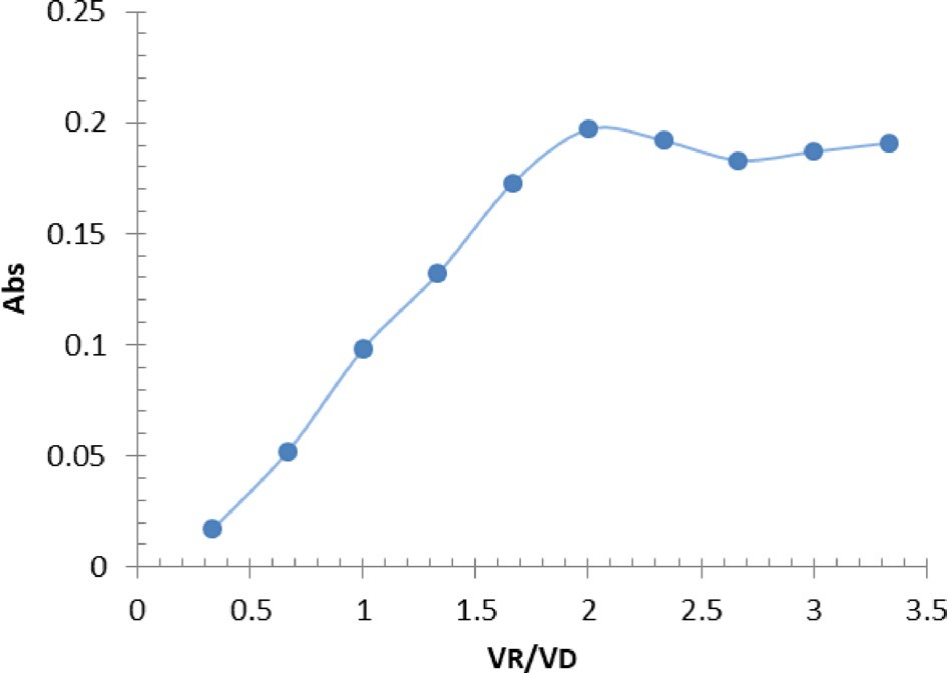
Molar ratio method.

The molar ratio method involves adding 0.6 ml of a standard FMD solution to a series of volumetric flasks, each containing 5.0 ml. Different volumes of a reagent solution ranging from 0.2 to 2 ml are then added, along with 0.5 ml of NaOH 0.1 M. The flasks are then filled to the mark with double-distilled water, and the absorbance is measured at 546.0 nm, using the blank reagent as a reference. The data presented in Fig. 5 indicates a ratio of 2:1.

### The suggested chemical reaction

*Ortho-*chloranil bonds strongly with chemical atoms having electron pairs, especially nitrogen^21^. *Ortho-*chloranil and nitrogen form a 2:1 complex (nitrogen: Ortho-chloranil), demonstrating the participation of the nitrogen atoms in the making of the complex. The complex displays a clear peak caused by charge transfer, which is distinct from the absorbance peaks of both the donor and acceptor molecules. The occurrence of this new peak indicates the creation of an n-π charge transfer (CT) complex. When this combination is exposed to visible or UV radiation, it undergoes further changes, resulting in the creation of two free radical ions: D + and A-^22^. NaOH expedites the quick synthesis of free radicals. The complexation reaction, which involves the transfer of charge and the production of free radicals, can be represented as follows:

Charge transfer complexes and free radicals form when *Ortho*-chloranil interacts with the hydroxyl group, is interesting in many chemical processes and analytical applications. This study highlights the importance of *Ortho*-chloranil as a π-acceptor reagent in studying complex formation mechanisms and free radical reactions.

### Applications

The procedure was applied to pharmaceutical preparations containing FMD 20 mg/Tablet.

#### Direct method

Three various concentrations (4, 12 and 20) μg/ml were used. The solution passed the identical procedures as the calibration curve and was assessed at a wavelength of 546.0 nm. The outcomes are displayed in Table 11, indicating the efficacy of the proposed approach in evaluating FMD in pharmaceutical formulation.

**Table 11:** Direct method.

Drug	Taken μg/ml	Found μg/ml	Average of Rec%	RSD%
Tablets (20 mg)	4	4.129	**99.353**	1.415
	12	11.709		1.131
	20	19.451		1.982

#### Standard additions

**Figure 6:**
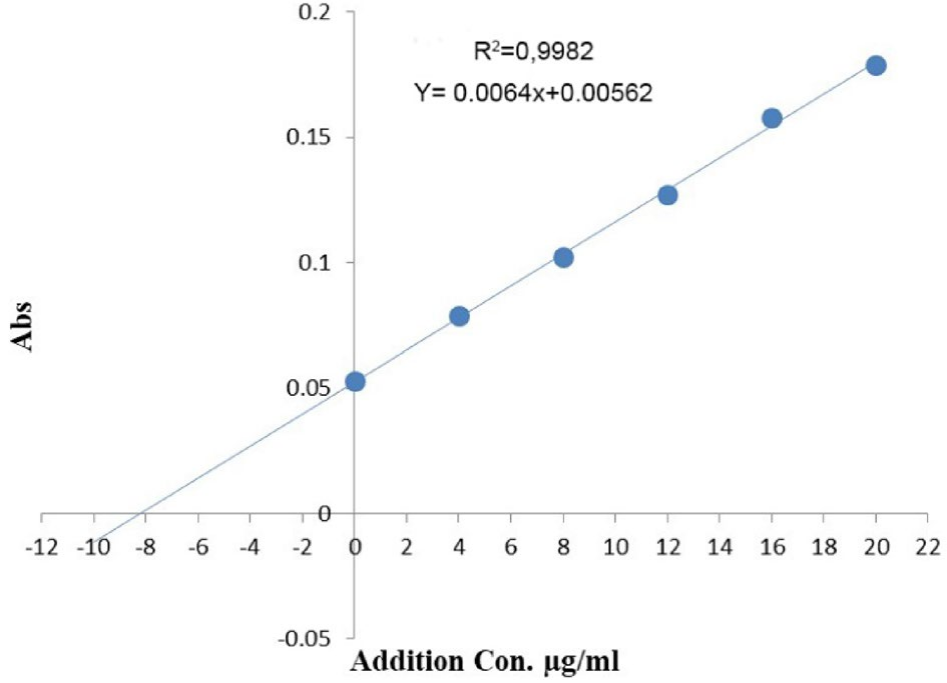
Standard addition method.

The standard additions method was applied in order to prove that the developed method is free of interference based on the concentration of the drug by taking 0.8 ml of pharmaceutical preparation solution at a concentration of 100 μg/ml of FMD to six 5.0 ml volumetric flasks, then adding an increasing volume of standard solution FMD (0.2–1.0) ml with the six bottles remaining without addition. After that, it was measured at 546.0 nm, like the calibration curve. Figure 6 and Table 12 illustrate typical addition results. Spectrophotometric and suggested approaches for famotidine determination in pharmaceutical formulations are compared in Table 13.

**Table 12:** Accuracy of standard addition method.

Famotidine tablets (20 mg)	
Taken µg/ ml	8
Found µg/ ml	8.101
Recovery%	101.1
RSD%	0.988

**Table 13:** Comparison of the proposed method with spectrophotometric methods.

No	Parameters	The method in this study	Method 1^22^	Method 2^23^	Method 3^24^
1	λ max nm	540.0	260	420	287
2	Beers law limit μg/ml	2.0—28	12.5–20	0.7–8.1	2–10
3	Colour	Violet	–	Yellow	–
4	Correlation coefficient	0.9977	0.920	0.9985	0.993
5	Sandell sensitivity μg/cm^2^	0.15625	–	–	–
6	Molar absorptivity L/mol cm	2159.648	–	2 × 10^4^	–
7	Average of Rec%	100.345	98–102	101.7	99.58
8	LOD (μg/ml )	0.304 μg/ml	–	0.7	0.087
9	LOQ (μg/ml )	1.471 μg/ml	–	–	0.264

## Conclusions

A fast and accurate method for assessment was developed by using a charge transfer complex reaction based on the method of reduction FMD with *Ortho-*chloranil and NaOH 0.1 M, and the complex formed had a violet color, was soluble in water, and was stable for at least 40 min at a wavelength of 546 nm (R^2^ = 0.9977). The standard calibration curve shows that the linear range is 2–28 μg/ml, the recovery is 99.56%, the value of the standard deviation was less than the mean 1.22%, the molar absorptivity is 2159.648 L mol^−1^ cm^−1^, and the Sandell Index is 0.15625 μg.cm^−2^.

## Data Availability

The data used and/or analyzed during the current study are available from the corresponding author upon reasonable request.
